# Risk factors for the recurrence of atrial fibrillation after catheter ablation: a meta-analysis

**DOI:** 10.1186/s43044-025-00605-7

**Published:** 2025-01-13

**Authors:** Gonghao Li, Yanli Zhao, Zhongxing Peng, Yunfeng Zhao

**Affiliations:** 1 Department of Cardiology, Lianyungang No 1 People’s Hospital, No. 6 East Zhenhua Road, Haizhou District, Lianyungang, 222061 Jiangsu China; 2 The First Affiliated Hospital of Kangda College of Nanjing Medical University, Lianyungang, China

**Keywords:** Atrial fibrillation, Recurrence, Risk factors, Catheter ablation

## Abstract

**Background:**

The rate at which atrial fibrillation (AF) patients experience a return of symptoms after catheter ablation is significant, and there are multiple risk factors involved. This research intends to perform a meta-analysis to explore the risk factors connected to the recurrence of AF in patients following catheter ablation.

**Methods:**

The PubMed, Cochrane Library, WOS, Embase, SinoMed, CNKI, Wanfang, and VIP databases were explored for studies from January 1, 2000 to August 10, 2021, and research meeting the established inclusion requirements was chosen. Two authors separately gathered details regarding the study structure. The strength of the link between various risk factors and AF returning after CA was evaluated using odds ratios. All statistical evaluations were conducted with RevMan5.3 software.

**Results:**

In total, 44 articles and 62,674 patients were included. The OR for AF recurrence in patients with diabetes was 2.04 compared with the reference group (95% CI 1.51–2.76, *p* < 0.00001); that of lower left ventricular ejection fraction was 1.38 (95% CI 1.25–1.52, *p* < 0.00001); that of female was 1.34 (95% CI 1.18–1.52, *p* < 0.00001); that of increased age was 1.03 (95% CI 1.02–1.04, *p* < 0.00001); that of persistent AF was 1.72 (95% CI 1.58–1.87, *p* < 0.00001); that of AF duration over 2 years was 1.17 (95% CI 1.08–1.26, *p* < 0.00001); that of increased left atrial diameter (LAD) was 1.12 (95% CI 1.08–1.17, *p* < 0.00001); that of larger left atrial volume index (LAVi) was 1.02 (95% CI 1.01–1.03, *p* < 0.00001); that of higher hs-CRP was 1.19 (95% CI 1.04–1.36, *p* = 0.04); that of early recurrence (ER) was 3.22 (95% CI 2.74–3.77, *p* < 0.00001); and that of long ablation duration was 1.00 (95% CI 0.98–1.02, *p* = 0.72). Heterogeneity and slight publication bias were observed for each factor.

**Conclusions:**

Evidence indicates that diabetes, low left ventricular ejection fraction, being female, older age, longer duration of atrial fibrillation, elevated high-sensitivity C-reactive protein levels, large left atrial dimension, large left atrial volume index, persistent atrial fibrillation, and exercise rehabilitation are factors that increase the chances of getting atrial fibrillation again after catheter ablation. However, the length of the ablation procedure does not relate to the recurrence of AF.

## Background

Atrial fibrillation (AF) is a frequent irregular heartbeat, and its occurrence greatly rises as people get older [1]. From a medical point of view, individuals identified with AF face a much greater chance of heart failure, stroke, and death when compared to those without AF [2]. Catheter ablation, which includes methods like radiofrequency ablation and cryoablation, has become a commonly suggested treatment choice for patients with symptomatic AF who do not find relief with regular antiarrhythmic drugs. This suggestion is especially relevant for paroxysmal AF cases. Notably, this advice is founded on objective clinical proof rather than personal viewpoint [3, 4]. The primary treatment aim of catheter ablation is to achieve circumferential pulmonary vein isolation, which is intended to stop AF by disrupting the electrical activities that trigger its development [5, 6]. However, past research has indicated that the success rate of circumferential pulmonary vein isolation in treating atrial fibrillation ranges from 50 to 80% [3]. Even with constant upgrades in ablation technology and progress in methods, many patients continue to have recurring AF, which requires more or ineffective ablations. Notably, studies show that in some instances, AF does not return even after the pulmonary veins are reconnected [7]. These results suggest that the return of AF following catheter ablation is influenced by several elements. A thorough understanding of the clinical indicators of AF return is essential for improving patient care after ablation. Hence, the main goal of this research is to explore the connection between different risk factors and the chances of AF returning after catheter ablation.

## Methods

### Database search

A comprehensive literature search was performed across several major databases, such as PubMed, Cochrane Library, WOS, Embase, SinoMed, CNKI, Wanfang, and VIP, utilizing the keywords “risk,” “recurrence,” and “atrial fibrillation,” utilizing the keywords “risk,” “recurrence,” and “atrial fibrillation.” We do not use the artificial intelligence (AI)-assisted technology in the production of our submissions.

### Study selection

This study aimed to investigate the relationship between various risk factors and the recurrence of AF following catheter ablation. To achieve this, we selected cohort and case–control studies that reported on one or both of the following outcomes: (1) Patients who met the defined diagnostic criteria [8], with no race, age, and gender, and (2) studies focused on identifying risk factors influencing postoperative AF recurrence after catheter ablation. Studies such as letters, editorials, or those lacking control groups or not reporting relevant outcomes were excluded. Furthermore, articles that did not include odds ratios (OR) with corresponding 95% confidence intervals (CI) or the methodology for calculating standard errors were omitted. Similarly, studies with endpoints unrelated to AF recurrence were not considered. Early recurrence (ER) of AF or atrial tachycardia was defined as events occurring within three months post-ablation, while AF duration was categorized by the length of time the arrhythmia had persisted since its initial diagnosis.

### Data extraction and quality assessment

The processes of literature retrieval, data extraction, and quality assessment were conducted independently by two authors based on predefined inclusion criteria. Any discrepancies between the authors were resolved through mutual consultation. The collected data included study design, patient demographics, details of the catheter ablation procedure, and methods used to detect atrial fibrillation recurrence. The quality of the studies selected for inclusion was assessed using the Newcastle–Ottawa scale (NOS) to ensure reliability [9].

### Statistical analyses

To assess the relationship between risk factors and AF recurrence after catheter ablation, multivariable-adjusted odds ratios (ORs) with 95% confidence intervals (CI) were used. ORs, along with their standard errors, were derived from the 95% CI and underwent logarithmic transformation to stabilize variance and normalize the data distribution. Heterogeneity across the included studies was measured using the Cochrane’s Q test [10] and the I^2^ test [11]. A random-effects model was utilized for the synthesis of the results, given that significant heterogeneity was indicated by an I^2^ value exceeding 50% [10]. The robustness of the results was confirmed by sensitivity analyses conducted by excluding individual studies on a case-by-case basis [12]. Funnel plot analysis was used to assess the potential publication bias. RevMan (Version 5.3; Cochrane Collaboration, Oxford, UK) was subjected to a meta-analysis and statistical analysis.

## Results

### Search results

**Fig. 1:**
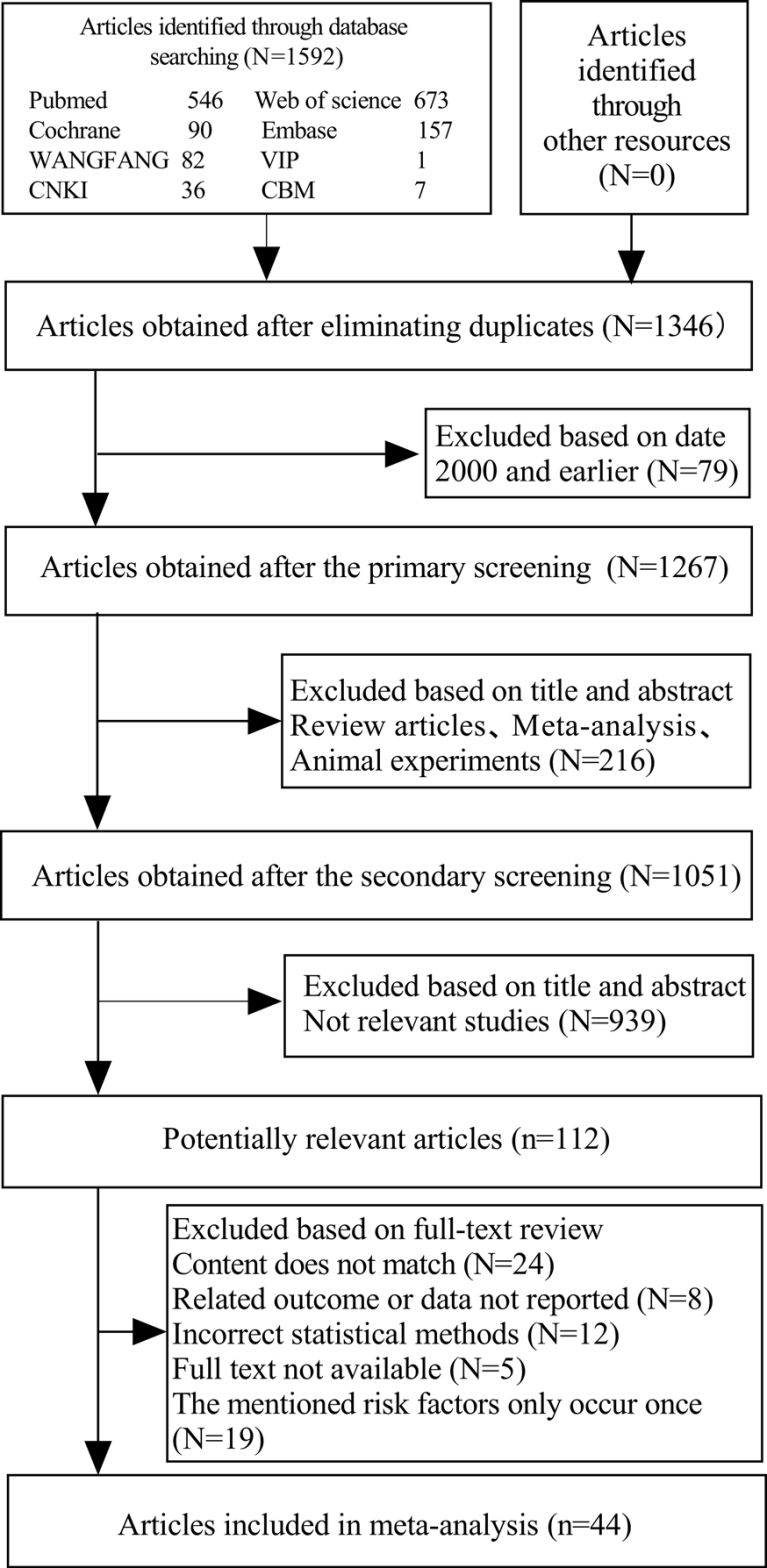
Study flow diagram

The graph in Fig. 1 gives a summary of the ways to find literature. To sum up, the first part includes searching a database that gets rid of repeat entries, yielding 1,346 possible studies. Next, we removed 790 studies that came out before the year 2000. The first screening looked at titles, abstracts, and types of literature, leading to the removal of 216 studies. After that, a closer look at the titles and abstracts of the studies left resulted in the elimination of 939 more studies, mainly because they did not connect to our research goals. A total of 112 research papers were examined, leading to the removal of 68 papers for different reasons: 24 papers did not match the research topic, 8 papers did not report their findings, 12 papers used wrong statistical techniques, 5 papers were unable to supply complete texts, and 19 papers had risk factors that relied on just one event. As a result, our meta-analysis consisted of 44 papers. 12 papers used wrong statistical techniques, 5 papers were unable to supply complete texts, and 19 papers had risk factors that relied on just one event. As a result, our meta-analysis consisted of 44 papers.

### Study characteristics and quality evaluation

Table 1 gives a summary of the features of the studies that are part of this meta-analysis. In all, the analysis covered 33 cohort studies and 11 case–control studies, involving 62,674 AF patients who received catheter ablation. The general quality of these studies was considered acceptable, with Newcastle–Ottawa scale (NOS) scores varying from 7 to 9.

**Table 1:** Characteristics of the included studies

References	Country	Patient number	Number of cases with AF recurrence	CA details	Mean follow-up months	Type of study	NOS	Risk factors involved
Koyama et al. [55]	Japan	186	72	RF-CPVI	6	cohort study	9 (4/2/3)	ER
Wokhlu et al. [15]	USA	774	277	RF-CPVI	36	cohort study	9 (4/2/3)	Diabetes, LAD, AF Types
Yang et al. [51]	China	138	80	RF-CPVI plus	9.5	cohort study	9 (4/2/3)	LAD
Yoshida et al. [30]	USA	79	13	RF-CPVI	6	cohort study	9 (4/2/3)	Age, Ablation time
Gu et al. [47]	China	150	36	RF-CPVI	22.9	cohort study	9 (4/2/3)	LAD
Cha et al. [16]	USA	368	97	RF-CPVI	12	cohort study	9 (4/2/3)	AF Types, CHF, Female
Richter et al. [52]	Austria	30	16	RF-CPVI	6	cohort study	9 (4/2/3)	hs-CRP
Roland Richard (2012)	Germany	202	111	RF-CPVI plus	56	cohort study	9 (4/2/3)	AF duration
Sotomi et al. [53]	Japan	392	40	RF-CPVI	32.4	case control study	7 (3/2/2)	hs-CRP
Morris et al. [23]	Germany	84	19	RF-CPVI	19.2	cohort study	9 (4/2/3)	CHF
Kalil et al. [48]	Brazil	102	33	RF-CPVI plus	8	cohort study	9 (4/2/3)	LAD
Park et al. [28]	Korea	576	80	RF-CPVI plus	13.1	cohort study	9 (4/2/3)	Age, ER
Deftereos et al. [18]	Greece	206	83	RF-CPVI	15	cohort study	9 (4/2/3)	LAD, CHF
Kumar et al. [38]	USA	124	49	RF-CPVI	11.8	case control study	8 (3/2/3)	AF Types
Kim et al. [37]	Korea	665	176	RF-CPVI plus	19.3	cohort study	9 (4/2/3)	AF Types
Bohó et al. [34]	Slovakia	205	15	CB-RFCA	34	case control study	8 (3/2/3)	AF Types
Teunissen et al. [40]	Netherlands	509	299	RF-CPVI	66	cohort study	8 (4/2/2)	AF Types, AF duration
Dallaglio et al. [17]	Spain	666	19	RF-CPVI	45	case control study	7 (3/2/2)	CHF, Female
Cabanelas et al. [49]	Spain	94	42	NA	NA	cohort study	7 (4/2/1)	LAD
Park et al. [43]	Korea	141	103	RF-CPVI	25	cohort study	9 (4/2/3)	AF duration, Age
Yingxian et al. [45]	China	55	30	NA	40	cohort study	9 (4/2/3)	AF duration
Deng et al. [19]	China	1410	365	RF-CPVI	20.7	case control study	8 (3/2/3)	AF Types, Age LAD, CHF
Carballo et al. [31]	Switzerland	195	101	RF-CPVI	6	cohort study	9 (4/2/3)	Ablation time, hs-CRP
Arora et al. [13]	USA	37,360	1964	NA	3	case control study	8 (3/2/3)	Diabetes female
Linhart et al. [39]	Spain	94	40	RF-CPVI	15	cohort study	8 (4/2/2)	AF Types
Kim et al. [26]	Korea	874	195	RF-CPVI plus	29.9	cohort study	8 (4/2/2)	Age, LAVI
Knecht et al. [42]	Switzerland	129	36	RF-CPVI	24	cohort study	9 (4/2/3)	AF duration
Bisbal et al. [33]	Spain	243	105	RF-CPVI CB-CPVI	43	cohort study	9 (4/2/3)	AF Types
Fredersdorf et al. [36]	Germany	122	24	RF-CPVI	51.1	cohort study	8 (4/2/2)	AF Types
Lambert et al. [24]	Czech Republic	61	43	RF-CPVI	19.5	cohort study	9 (4/2/3)	Female
Stabile et al. [32]	Italy	2519	571	RF-CPVI	46.2	cohort study	9 (4/2/3)	Ablation time, AF duration
Wang et al. [14]	USA	351	134	RF-CPVI	29.5	case control study	8 (3/2/3)	Diabetes, AF Types
Deng et al. [20]	China	1407	365	RF-CPVI CB-CPVI	20.7	cohort study	9 (4/2/3)	AF Types, LAD, Age CHF, ER
Deng et al. [25]	China	1410	365	CB-CPVI	20.7	cohort study	9 (4/2/3)	AF Types, LAD, Age hs-CRP, ER
Sabina Istratoaie (2019)	Romania	80	30	RF-CPVI	14	case control study	8 (3/2/3)	LAVI
Shang et al. [50]	China	100	31	CB-CPVI	13.4	cohort study	9 (4/2/3)	LAD
Donnellan et al. [21]	USA	591	271	RF-CPVI plus	32	cohort study	9 (4/2/3)	AF Types, LAVI
Donnellan et al. [35]	USA	267	87	RF-CPVI plus	29	cohort study	9 (4/2/3)	CHF
Donnellan et al. [46]	USA	478	220	RF-CPVI	29	cohort study	9 (4/2/3)	LAD
Yu et al. [41]	Korea	1005	313	RF-CPVI plus	24	cohort study	9 (4/2/3)	AF Types
Lili et al. [27]	China	120	38	CB-CPVI	12	case control study	8 (3/2/3)	LAD, Age,hs-CRP, AF duration
Shchetynska et al. [29]	Germany	205	88	NA	21	case control study	8 (3/2/3)	Age
Kaneko et al. [22]	Japan	568	91	RF-CPVI CB-CPVI	14	case control study	8 (3/2/3)	CHF
Hodges et al. [88]	Denmark	7339	2801	NA	NA	cohort study	9 (4/2/3)	ER

CB, cry balloon; LAD, left atrial dimension; LAVI, left atrial volume indexed; PV, Pulmonary Vein

### Risk factors and AF recurrence

**Fig. 2:**
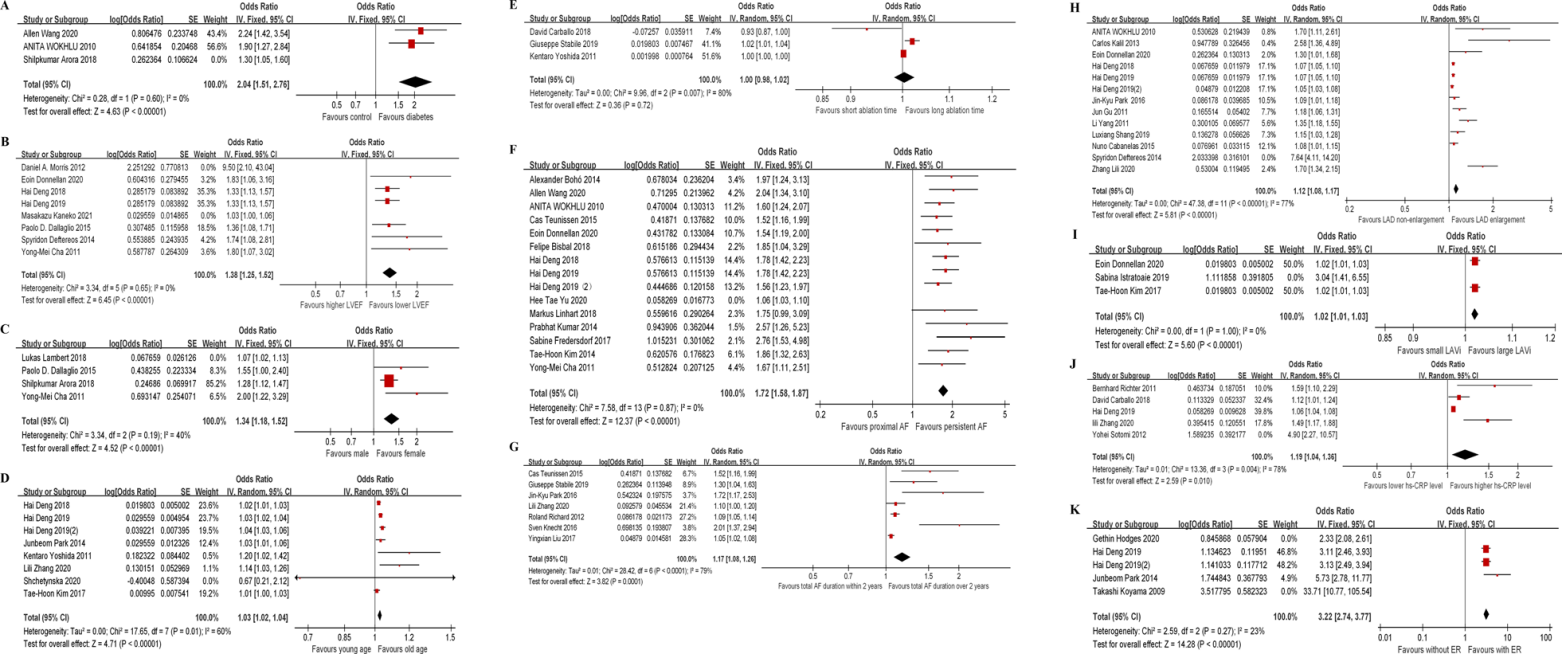
Forest plot for risk factors. **A** Forest plot for diabetes. **B** Forest plot for heart failure. **C** Forest plot for gender. **D** Forest plot for age. **E** Forest plot for ablation duration. **F** Forest plot for AF types. **G** Forest plot for AF duration. **H** Forest plot for LAD. **I** Forest plot for LAVi. **J** Forest plot for hs-CRP. **K** Forest plot for ER. AF: atrial fibrillation; LAD: left atrial diameter; LAVi: left atrial volume index; CRP: C-reaction protein

In the section discussing the meta-analysis of the relationship between diabetes and the risk of AF recurrence, the results from three studies are highlighted [13–15]. Overall, as shown in Fig. 2A indicates that patients with diabetes exhibited a significantly elevated risk for AF recurrence in comparison to the control group (OR 2.04; 95% CI 1.51–2.76; *p* < 0.00001) without heterogeneity (*p* = 0.60, I^2^ = 0%). To investigate the potential sources of heterogeneity within the pooled data, a sensitivity analysis was performed by sequentially excluding one study at a time. This process revealed that the heterogeneity observed in the analysis of diabetes was predominantly attributed to the findings reported by Shilpkumar et al.

In the meta-analysis examining the link between heart failure and the likelihood of AF recurrence across eight studies [16–23]. Overall, patients with heart failure exhibited a significantly higher risk of developing AF recurrence in comparison to the control group (OR 1.38; 95% CI 1.25–1.52; *p* < 0.00001) without heterogeneity (*p* = 0.65, I^2^ = 0%). In a bid to identify the sources of any likely differences within the total data, a careful sensitivity examination was carried out. This process included the step-by-step removal of one trial at a time. The results from this examination showed that the main factors leading to the seen differences in heart failure were the research shared by Daniel et al. and Masakazu et al., as shown in Fig. 2B.

Data pertaining to sex demographics were reported across four studies [13, 16, 17, 24], and Fig. 2C illustrates the findings derived from a fixed-effects model that aggregates the OR for female participants. Overall, when compared to the control group, female patients experienced a moderately increased risk for developing AF recurrence (OR 1.34; 95% CI 1.18–1.52; *p* < 0.00001) with substantial heterogeneity (*p* = 0.19, I^2^ = 40%). Upon further scrutiny to identify the source of this heterogeneity, it became evident that the variance was predominantly attributable to the findings reported by Daniel et al. and Lukas et al. This observation highlights the need to take into account the role of sex-related factors when evaluating the danger of AF coming back.

In the meta-analysis examining the impact of age on the risk of AF recurrence across eight studies [19, 20, 25–30], the results indicate that older patients face a significantly heightened risk of AF recurrence compared to the control group. Overall, compared with the control group, aging patients experienced a significantly increased risk for developing AF recurrence (OR 1.03; 95% CI 1.02–1.04;* p* < 0.00001) with substantial heterogeneity (*p* = 0.01, I^2^ = 60%, Fig. 2D). This heterogeneity suggests that factors beyond age alone may be influencing the risk of AF recurrence, and further investigation is warranted to understand the complex interplay between age and other potential risk factors.

Three studies [30–32] provided data on ablation time, and Fig. 2E presents the analysis of this data. Figure 2E shows that patients with longer ablation duration may not have a decreased the risk for developing AF recurrence (HR 1.00; 95% CI 0.98–1.02; *p* = 0.72). This analysis implies that the length of ablation treatment may not be a significant predictor of AF recurrence, and other factors may be more influential in determining the outcome.

A total of 15 studies reported AF type data [14–16, 19, 20, 25, 33–41]. Figure 2F presents the findings of the fixed-effects model, which combines the OR of a persistent AF. The persistent AF patients exhibited a greater risk of recurrence after the first ablation (OR 1.72; 95% CI 1.58–1.87; *p* < 0.00001) without substantial heterogeneity (*p* = 0.87, I^2^ = 0%). The analysis indicated that heterogeneity in AF type was mainly contributed by reports by Hee et al.

In the meta-analysis investigating the correlation between the duration of AF and the risk of AF recurrence across seven studies [27, 32, 40, 42–45]. Figure 2G illustrates that patients with an AF history exceeding two years are at a significantly higher risk of recurrence. Patients with over 2 years of AF duration experienced a significantly increased risk for developing recurrence (OR 1.17; 95% CI 1.08–1.26; *p* < 0.00001), with substantial heterogeneity (*p* < 0.0001, I^2^ = 79%).

One test for each round was left out and utilized to check the reason for the differences in the combined information. To find the cause of this variation, a sensitivity study was performed by removing one research at a time in order. The outcomes of this study showed that the differences in AF duration were mainly due to the results presented by Cas et al., Jin et al., and Sven et al. This implies that the effect of AF duration on the risk of returning may differ greatly based on the particular patient groups and methods applied in these researches.

Meta-analysis of LAD and risk of AF recurrence in 13 studies [15, 18–20, 25, 27, 43, 46–51]. Overall, patients with an enlarged LAD experienced an increased risk for developing AF recurrence (OR 1.12; 95% CI 1.08–1.17; *p* < 0.00001), with substantial heterogeneity (*p* < 0.00001, I^2^ = 77%). Spyridon et al. was excluded because it was the main reason for the heterogeneity (Fig. 2H).

A total of 3 studies reported LAVi data [26, 32, 35]. Figure 2 I demonstrates that patients with larger LAVi exhibited a significantly elevated risk of developing AF recurrence (HR 1.02; 95% CI 1.01–1.03; *p* < 0.00001), without substantial heterogeneity (*p* = 1.00, I^2^ = 0%). Analyses showed that the heterogeneity of LAVi was mainly from the reports by Sabina et al.

Meta-analysis of hs-CRP and risk of AF recurrence in 5 studies [25, 27, 31, 52, 53]. Figure 2J demonstrates that patients with higher baseline hs-CRP had a significantly increased risk of developing AF recurrence (HR 1.19; 95% CI 1.04–1.36; *p* = 0.010), with substantial heterogeneity (*p* = 0.004, I^2^ = 78%). The analysis showed that the heterogeneity of hs-CRP mainly came from the report of Yohei et al.

A total of five studies reported ER data [20, 25, 28, 54, 55]. Overall, the study found that patients who with ER had a significantly higher risk of recurrent atrial fibrillation compared to the control group (HR 3.22; 95% CI 2.74–3.77; *p* < 0.00001). It is, however, imperative to recognize the notable heterogeneity present in the study outcomes (*p* = 0.27, I^2^ = 23%). After carrying out a thorough examination to identify the cause of this variation, it was clear that the differences regarding early recurrence were mainly affected by the findings from Gethin et al. and Takashi et al. This finding highlights the significance of taking into account the methodological and demographic distinctions between studies when analyzing how early recurrence affects the likelihood of AF recurrence.

### Publication bias

To verify the sources of heterogeneity in the merged data, one experiment was omitted in each round.

**Fig. 3:**
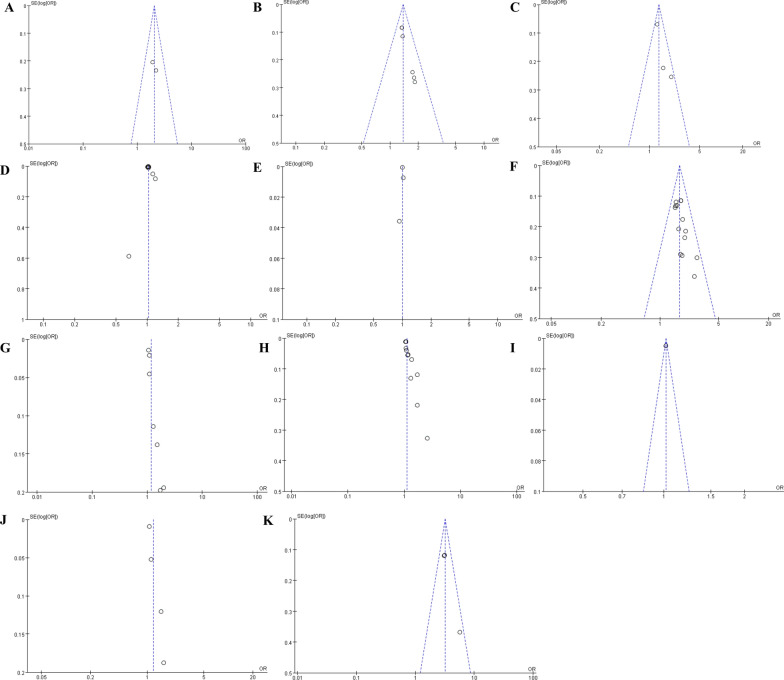
Funnel plot for risk factors. **A** Funnel plot for diabetes. **B** Funnel plot for heart failure. **C** Funnel plot for gender. **D** Funnel plot for age. **E** Funnel plot for ablation duration. **F** Funnel plot for AF types. **G** Funnel plot for AF duration. **H** Funnel plot for LAD. **I** Funnel plot for LAVi. **J** Funnel plot for hs-CRP. **K** Funnel plot for ER. AF: atrial fibrillation; LAD: left atrial diameter; LAVi: left atrial volume index; CRP: C-reaction protein

Figure 3 shows the funnel plot of the meta-analysis examining the association between risk factors after catheter ablation and recurrence. A visual inspection of the chart reveals a symmetrical shape, indicating a low risk of publication bias.

## Discussion

AF is the most common form of heart rhythm issue, recognized for its gradual development that usually leads to regular recurrences. Catheter ablation has emerged as an important treatment method for handling AF, particularly for patients who do not gain enough relief from antiarrhythmic medications, ultimately enhancing their general quality of life [56]. Despite the newest enhancements in surgical and ablation techniques, such as the use of contact force catheters and advanced mapping technologies, repeat AF is still seen in about 30% to 60% of patients after catheter ablation [57–59]. Our meta-analysis uncovered 11 distinct risk factors associated with the recurrence of AF following catheter ablation, encompassing diabetes, heart failure, gender, age, AF classification, duration of AF, left atrial diameter (LAD), left atrial volume index (LAVi), high-sensitivity C-reactive protein (hs-CRP), and early recurrence (ER). Notably, the risk associated with these factors was quantified, revealing that the risk was increased by 2.04-fold for diabetes, 1.38-fold for heart failure, 1.34-fold for female gender, 1.03-fold for advancing age, 1.72-fold for persistent AF, 1.17-fold for AF duration exceeding two years, 1.12-fold for enlarged LAD, 1.02-fold for increased LAVi, 1.19-fold for elevated hs-CRP levels, and 3.22-fold for ER, when compared to the control group. On the other hand, the length of ablation did not show a meaningful effect on the rates of recurrence.

The relationship between diabetes and the return of AF has been a well-studied subject, yet the findings of different studies frequently vary [60–62]. Our analysis confirms that the risk of recurrence of AF in diabetic patients is significantly increased, which may be due to atrial remodeling caused by diabetes, potentially undermining the long-term success of catheter ablation.

AF is the most common arrhythmia among individuals with heart failure, and having both these issues at the same time can significantly worsen the well-being of patients and increase the dangers related to illness and death [63]. In a previous meta-analysis [64], a higher prevalence of AF recurrence in patients with heart failure post-catheter ablation compared to those without heart failure. The effectiveness of catheter ablation in treating AF episodes in individuals with heart failure is frequently reduced, as heart failure encourages the development and worsening of AF by changing the structure and electrical activity of the atria. Moreover, during catheter ablation treatments, patients with heart failure may face more significant technical difficulties because of changed anatomical connections and higher LAD.

Gender disparities in the recurrence of AF are well-documented, with female patients exhibiting thinner atrial sinus walls and a higher prevalence of non-pulmonary vein triggers compared to their male counterparts [65–67]. Even though women are less prone to have catheter ablation and atrial fibrillation is more common in men, research has indicated that women have a greater chance of having a return of symptoms in the first year after electrical cardioversion. Our review verified a notably higher recurrence rate in female patients when compared to male patients, consistent with earlier studies [25, 68].

As stated earlier, handling AF in older individuals continues to be difficult because of various possible complications [69]. Our findings indicate that advancing age is linked to the return of AF after CA, aligning with an earlier investigation [70]. Elderly patients with AF are more prone to have non-PV lesions, which are usually found in the superior vena cava, left atrial free wall, crista terminalis, coronary sinus foramen, ligamentum of Marshall, left atrial appendage, and atrial septum [71]. Growing older is also related to changes in the structure and electrical activity of the atrium.

In our research, we noticed that increasing the ablation duration did not substantially lower the AF recurrence rate. This result aligns with the findings of Chen et al. and Yoga et al., who stated that high-power, brief ablation methods are linked to reduced recurrence rates when compared to standard ablation procedures [72, 73]. These strong methods have been proven to be safe, effective in shortening overall procedure time, and provide similar long-term success in preventing AF from returning. It is important to acknowledge the possible negative effects of too much ablation, which can lead to weakened atrial conduction and a higher chance of blood clot-related issues.

Multiple earlier studies have verified that persistent AF is a risk factor for recurrent AF [74–76].

In contrast to paroxysmal atrial fibrillation, the group of persistent atrial fibrillation includes a varied range of atrial fibrillation development and changes. Additionally, the length of time atrial fibrillation lasts is a risk factor for its return. The longer atrial fibrillation continues, the greater the chances of it coming back after catheter ablation. Atrial fibrillation is a worsening condition marked by changes in the stretching of the atria, swelling, and harm to atrial cells. This harm leads to scarring and fibrosis [15, 77]. As the changes in structure and electrical patterns build up in individuals with long-lasting AF, the chances of getting back and keeping a normal heartbeat grow more difficult. In our research, we found that individuals with AF persisting for over two years have a higher chance of it coming back. Thus, applying catheter ablation early may improve the success of the treatment by addressing the initial phases of the AF development, which not only lowers the chances of atrial tachyarrhythmias returning but also leads to more favorable results than procedures done at a later time.

Research has established that a LAD exceeding 45 mm marks a critical threshold [78], and is often linked to a heightened risk of AF recurrence [79]. The main causes of AF returning in people with larger atrial sizes are linked to changes in electrical and structural aspects. A rise in atrial size leads to greater structural difficulty, which makes it harder to accomplish complete ablation, thus allowing the pulmonary veins to reconnect [80]. LAD is paralleled by left atrial volume, which more accurately reflects left atrial remodeling than linear diameter measurements. The left atrial volume index (LAVi) provides a clearer evaluation of left atrial growth and has been recognized as an indicator of AF return after catheter ablation [81]. Our results confirm that LAVi acts as a reliable measure for assessing the chance of AF returning, consistent with earlier academic research [81, 82].

Fibrosis and inflammation are intricately linked and share common pathogenic pathways. High levels of CRP signify systemic inflammation, which could lead to electrophysiological and structural changes within the atrial chambers. A growing body of research suggests a possible link between inflammation and both the initiation and perpetuation of AF [83, 84]. CRP was associated with the emergence or recurrence of AF [85]. Our study combining various analyses shows that high initial levels of high-sensitivity C-reactive protein (hs-CRP) are notably linked to the return of AF after catheter ablation, a result that enhances the existing understanding of the relationship between inflammation and AF recurrence and aligns with earlier research findings [86, 87].

Our research results indicate that individuals who had early recurrence of their condition had more than double the chance of experiencing a later return, which matches the results of an earlier study [88]. Tao et al.'s research identified early recurrence in individuals with enlarged LAD [89]. It is believed that an early recurrence might interfere with the remodeling process after the ablation, resulting in an incomplete reshaping of the atrial structure, which could increase the likelihood of future AF recurrences.

It is essential to recognize that our research has some limitations that should be considered when analyzing the findings. Firstly, the funnel chart shows a possibility of publication bias. Secondly, a few studies were left out because they could not be reproduced, which might have led to bias. Finally, the varying follow-up times, ranging from 3 to 66 months, may also contribute to heterogeneity.

## Conclusions

This research outlines the connection between different risk factors and the return of AF following catheter ablation. While the specific ways these risk factors connect to AF recurrence are not completely clear, they might be due to multiple causes. Some risk factors, like age and gender, cannot be changed, whereas others, such as diabetes, inflammation, reduced heart function, and ongoing atrial fibrillation, can be altered and act as goals for prompt action. This thorough approach involves controlling blood sugar levels, treating heart failure, and quickly addressing infections.

In clinical practice, it is essential to incorporate risk factor modification into the assessment of patients who may have atrial fibrillation. This assessment should include the aforementioned risk factors. Thorough management of these risk factors might improve the effectiveness of ablation treatments.

## Data Availability

The study’s data are available for further analysis upon the reasonable request of the corresponding author.

## Abbreviations

AF: Atrial fibrillation
RFA: Radiofrequency ablation
CPVI: Circumferential pulmonary vein isolation
CA: Catheter ablation
NOS: Newcastle–Ottawa scale
SE: Standard errors
OR: Odds ratio
LVEF: Left ventricular ejection fraction
LAD: Left atrial diameter
LAVi: Left atrial volume index
ER: Early recurrence
